# Corrosion of dental alloys in artificial saliva with *Streptococcus mutans*

**DOI:** 10.1371/journal.pone.0174440

**Published:** 2017-03-28

**Authors:** Chunhui Lu, Yuanli Zheng, Qun Zhong

**Affiliations:** 1 Yongjia Clinic, Shanghai Stomatological Hospital, Fudan University, Shanghai, People's Republic of China; 2 Stomatology Special Consultation Clinic, Ninth People's Hospital, Shanghai Jiao Tong University, School of Medicine, Shanghai Key Laboratory of Stomatology, Shanghai, People's Republic of China; University of Akron, UNITED STATES

## Abstract

A comparative study of the corrosion resistance of CoCr and NiCr alloys in artificial saliva (AS) containing tryptic soy broth (Solution 1) and *Streptococcus mutans* (*S*. *mutans*) species (Solution 2) was performed by electrochemical methods, including open circuit potential measurements, impedance spectroscopy, and potentiodynamic polarization. The adherence of *S*. *mutans* to the NiCr and CoCr alloy surfaces immersed in Solution 2 for 24 h was verified by scanning electron microscopy, while the results of electrochemical impedance spectroscopy confirmed the importance of biofilm formation for the corrosion process. The R(QR) equivalent circuit was successfully used to fit the data obtained for the AS mixture without *S*. *mutans*, while the R(Q(R(QR))) circuit was found to be more suitable for describing the biofilm properties after treatment with the AS containing *S*. *mutans* species. In addition, a negative shift of the open circuit potential with immersion time was observed for all samples regardless of the solution type. Both alloys exhibited higher charge transfer resistance after treatment with Solution 2, and lower corrosion current densities were detected for all samples in the presence of *S*. *mutans*. The obtained results suggest that the biofilm formation observed after 24 h of exposure to *S*. *mutans* bacteria might enhance the corrosion resistance of the studied samples by creating physical barriers that prevented oxygen interactions with the metal surfaces.

## Introduction

Cobalt–chromium (CoCr) and nickel–chromium (NiCr) alloys are widely used in dental prostheses such as metal plates for complete dentures and frameworks for partial dentures, crowns, and bridges [1]. It is very important to study the corrosion characteristics of dental metals and alloys because their corrosion in the oral environment may adversely affect material biocompatibility and mechanical integrity, and lead to aesthetic loss of dental restorations, health hazards, and physical weakness. Serious concerns about the biocompatibility of dental alloys, related to the corrosion-induced metal ion release process, have been expressed [2]. Therefore, the investigation of the corrosion behavior of CoCr and NiCr alloys is essential for better understanding of their biocompatibility properties [3, 4].

The corrosion resistance of an alloy depends not only on its material properties, but also on its interactions with the surroundings [5]. The corrosion behavior of dental alloys can be affected by the oral environment (which may contain saliva, dental plaque, bacteria, and gastric acid reflux) as well as by the acidity and oxygen levels. The oral environment is warm and humid and continually experiences temperature fluctuations, while the moderate pH and temperature levels together with the abundance of nutrients create the ideal conditions for microorganism growth and development [6–8]. Microbiology-related corrosion (observed in the medical industry for many years) is defined as the deterioration of the metal surface due to the direct or indirect activity of living microorganisms. However, the exact mechanism describing the effect produced by the presence of bacteria and their byproducts on the corrosion behavior of metallic dental materials is currently unknown [9].

It has been previously shown that the microbial metabolic activities in complex biological systems can affect the pitting corrosion behavior of stainless steels [10]. Owing to the biofilm formation on metal surfaces and related effects, elucidating the interactions between dental materials and various bacterial species is very important for improving their corrosion properties.

The presence of *Desulfotomaculum nigrificans* sulfate reducing bacteria (SRB) was found to significantly affect the corrosion behaviour of Co-Cr-Mo and Ti-6Al-4V alloys [11], while *Streptococcus mutans* (*S*. *mutans*) and *Streptococcus sanguinis* species (which easily created biofilms on teeth surfaces) were able to corrode various orthodontic SUS appliances [12].

The mechanisms of microbially induced corrosion and the related corrosion inhibition process have not been elucidated in sufficient detail because of the inability to link them to a single biochemical reaction or specific microbial species or groups [13]. Thus, *S*. *mutans* bacteria were selected for investigation in this study because of their extensive presence in the oral environment of almost all people. In addition, *S*. *mutans* species grown under standard conditions are characterized by the abilities to produce a distinctive colonial morphology in any sucrose-containing medium (such as *mitis salivarius agar*), synthesize extracellular polysaccharides from sucrose, and undergo cell-to-cell aggregation when mixed with sucrose or dextran. These unique features of *S*. *mutans* may enhance its colonization activity in the oral cavity [9,14].

The purpose of this study was to investigate the early-stage microbiology-related corrosion behavior of NiCr and CoCr alloys in the presence of *S*. *mutans* by various electrochemical methods. The novelty of the conducted research is represented by the observed effect of the oral biofilms composed of *S*. *mutans* on the simultaneous corrosion behavior of the studied alloys in artificial saliva.

## Materials and methods

### Materials

#### Sample preparation

CoCr and NiCr alloys were acquired from Dental Alloys USA Inc. and Kennametal Stellite, respectively. Their compositions (listed in Table 1) were provided by the manufacturers.

**Table 1:** Chemical compositions of the utilized dental alloys (wt.%).

Alloy	Ni	Co	Cr	Mo	Mn+Si+Fe	Other
**NiCr (Kennametal Stellite, CHINA)**	64	–	22.5	9.5	–	<4
**CoCr (Dental Alloys, USA)**	–	61	28	5	3	<3

Sheet-like material samples with dimensions of 10 mm × 10 mm × 2 mm were prepared in accordance with the manufacturers’ recommendations using a laboratory lost wax technique [15]. Before corrosion testing, all samples were ground with 180-, 320-, and 600-grit Si carbide abrasive papers and then polished with 9-, 3-, and 0.05-μm diamond suspensions (Buehler, Germany) to produce mirror-like surfaces. A brass wire was welded to the specimen’s back side. The back and sides of each sample were embedded in a piece of epoxy resin with an area of 1 cm^2^, leaving the polished surface exposed. Each sample was cleaned ultrasonically in distilled water and ethanol for 2 min. To remove possible contaminations, the prepared test samples were exposed to ultraviolet light for 1 h.

#### Growth of *Streptococcus mutans* species

The microorganisms used in this study were *S*. *mutans* (UA159). Their primary colonizer was taken directly from an experimental room. *S*. *mutans* species were resuscitated from a frozen stock culture and incubated on plates at 37°C for 20–24 h in an atmosphere consisting of 5% CO_2_ and 95% N_2_ gases. Gram stain and colony morphology studies were conducted to confirm the purity of the grown *S*. *mutans* bacteria, which were collected from the incubating plates and placed in the tryptic soy broth (TSB) solution with the composition listed in Table 2. This solution had been previously sterilized in an autoclave (ES-315, TOMY, Japan) for 20 min at a temperature of 121°C and pressure of 15 psi.

**Table 2:** A composition of the TSB (g/L) solution utilized in this study.

Substance	Weight
**Pancreatic digest of casein (tryptone)**	17.0
**Papaic digest of soy meat (polypeptone)**	3.0
**Sodium chloride (NaCl)**	5.0
**Dipotassium phosphate (K**_**2**_**HPO**_**4**_**)**	2.5
**Yeast extract powder**	1.0
**Dextrose**	2.5

#### Electrolyte preparation

The composition of the artificial saliva (AS) solution utilized in this work is summarized in Table 3. To reduce contamination during preparation, the AS was sterilized by autoclaving for 20 min at a temperature of 121°C and pressure of 15 psi. Solution 1 was prepared from the mixture of the sterilized AS and TSB solutions with the AS:TSB volume ratio of 10:1, while Solution 2 was obtained from the sterilized AS mixed with *S*. *mutans* species at a volume ratio of 10:1. The *S*. *mutans* concentration of 10^8^ CFU/mL corresponded to the absorbance intensity of 1.0 obtained at a wavelength of 540 nm. Before electrochemical measurements the samples were immersed in Solution 1 or 2 for 24 h.

**Table 3:** A composition of the AS (g/L) solution obtained at pH = 6.8.

KCl	NaCl	MgCl_2_^.^6H_2_O	CaCl_2_^.^2H_2_O	Polypeptone	NaF	KH_2_PO_4_	K_2_HPO_4_
1.3	0.1	0.05	0.1	0.5	0.000025	0.027	0.035

### Methods

#### Electrochemical studies

A three-electrode electrochemical cell was utilized in all experiments. A saturated Ag/AgCl electrode was used as the reference electrode, a Pt electrode was used as the counter electrode, and the alloy samples were utilized as the working electrodes. Electrochemical measurements were conducted at a constant temperature of 37°C using a Princeton Applied Research electrochemical station (Parstat 2273, Ametek, Princeton, USA) controlled by a personal computer equipped with the PowerSuite software (Ametek, Princeton Applied Research). The samples treated with Solutions 1 and 2 were studied by conducting open circuit potential (OCP), electrochemical impedance spectroscopy (EIS), and potentiodynamic polarization measurements. Corrosion parameters including OCPs, corrosion current densities (icorr), and charge transfer resistance values (R_ct_) obtained from the electrochemical measurements were used to evaluate the corrosion resistance of the studied alloys. Three specimens were utilized during each corrosion testing procedure.

OCPs were measured within 1 h after the sample immersion in the electrolyte solution (the related potential–time curves were recorded at 2-s intervals). EIS measurements were performed in the frequency range between 10^5^ Hz and 10^−2^ Hz at an applied voltage amplitude of 10 mV after the OCP studies. R_ct_ values were determined from the equivalent circuits estimated by analyzing the plots obtained with the ZSimpWin software (Princeton Applied Research). Potentiodynamic polarization curves were recorded in the the scan range between -500 mV and +1600 mV (vs OCP) at a scanning rate of 1 mV/s [16]. I_corr_ values were determined using the curve-fitting routine of the PowerSuite software. All potentials in this study are reported with respect to the saturated Ag/AgCl electrode.

#### Surface analysis

To confirm the presence of bacteria on the metal surface and investigate the effect of the surface morphology on the alloy properties, the dental alloy samples treated with Solution 2 for 24 h were studied by scanning electron microscopy (SEM; JEOL JSM 6400, Tokyo, Japan). After incubation of *S*. *mutans* cells at 37°C for 24 h in microplate wells, a biofilm was formed on the sample surface. The specimens were first fixed with a 2.5% glutaraldehyde solution in phosphate buffer (pH = 7.0) for more than 4 h, washed three times in the phosphate buffer, post-fixed with a 1% OsO_4_ solution in phosphate buffer (pH = 7.0) for 1 h, and washed again three times in the phosphate buffer. Afterwards, the specimens were dehydrated with graded ethanol solutions, critical-point dried, coated with a Pd–Pt layer, and examined by SEM.

#### Statistical analysis

All corrosion tests were repeated until at least three similar results were obtained. The one-way analysis of variance (ANOVA) technique with a statistical significance level set to p = 0.05 was used (SPSS software, v11.5, USA).

## Results

### OCP measurements

**Fig 1:**
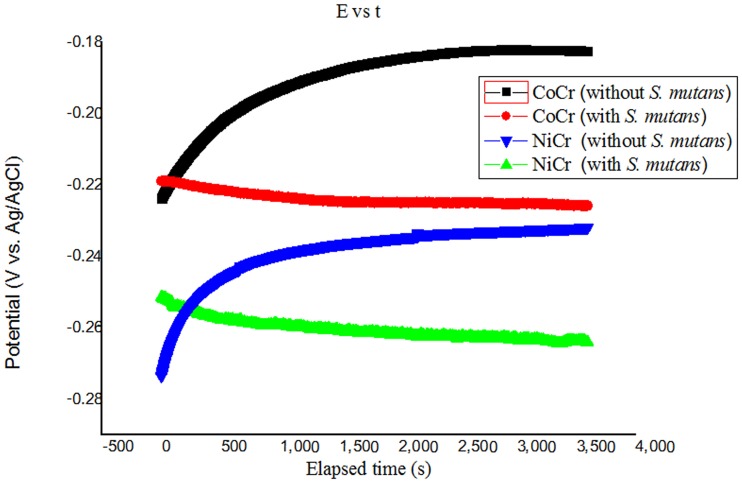
OCP values obtained for CoCr and NiCr alloys treated in Solutions 1 and 2 at 37°C.

The OCP values obtained for CoCr and NiCr alloys treated in both solutions at 37°C are plotted as functions of the immersion time in Fig 1. The OCPs of CoCr alloy treated with Solutions 1 and 2 for 1 h were stabilized at levels of -0.175 V and -0.237 V (vs. Ag/AgCl) respectively. Similarly, the OCPs of NiCr alloy were stabilized after 1 h of treatment with Solutions 1 and 2 at magnitudes of –0.244 V and –0.274 V (vs. Ag/AgCl), respectively. Hence, the OCPs of both alloys decreased after their treatment with the AS containing *S*. *mutans* species (the observed potential drops were 0.04 V (vs. Ag/AgCl) for CoCr alloy and 0.03 V (vs. Ag/AgCl) for NiCr alloy.Laurent et al [16] reported similar results for the OCP of a non-precious alloy immersed in AS solution.

### EIS measurements

**Fig 2:**
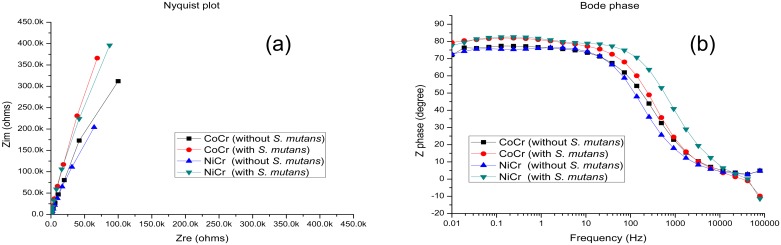
(a) Nyquist and (b) Bode plots obtained for CoCr and NiCr alloys treated in Solutions 1 and 2 at 37°C.

Fig 2 shows the EIS data obtained for the alloy samples immersed in Solutions 1 and 2 for 24 h. The Nyquist diagram (Fig 2a) displays a single capacitive arc in the complex plane with a diameter increasing in the presence of *S*. *mutans* (higher arc radii generally result in higher magnitudes of R_ct_). The Bode diagram (Fig 2b) shows that all of the treated samples exhibited high impedance corresponding to capacitive behavior. The phase angle in the 10^−2^–10^2^ Hz frequency range was close to 80°, and the phase change at higher frequencies was approximately 0°, indicating that the main resistance was ohmic (similar results were reported by Liu et al. [17]). All samples were in the passive state after the exposure to the simulated oral environment. Only one time constant was measured for the dental alloy samples that were not exposed to *S*. *mutans*, whereas two different time constants were obtained for the samples exposed to Solution 2 (their values were estimated by fitting the corresponding Bode diagram).

**Fig 3:**
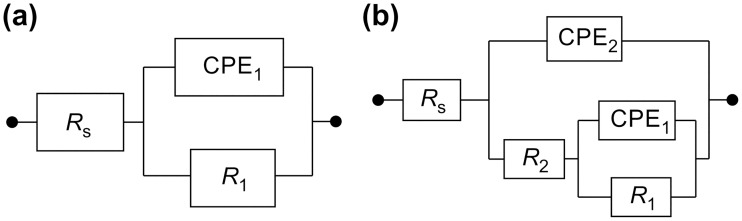
Equivalent circuits utilized for fitting the EIS results obtained in the (a) absence and (b) presence of *S*. *mutans* species. R_s_: solution resistance in the vicinity of the test sample, R_1_: charge-transfer resistance of CoCr and NiCr alloys, CPE_1_: double-layer capacitance of CoCr and NiCr alloys, R_2_: resistance of the biofilm binding layer, CPE_2_: capacitance of the biofilm binding layer.

It is typically difficult to design new equivalent circuits for complex systems such as the electrodes partially covered with biofilms. The EIS results obtained in this study were fitted with well-known equivalent circuits [18] using the ZSimpWin software (see Fig 3). The R(QR) circuit (Fig 3a) was used to fit the data obtained for the samples immersed in the sterile medium, and the R(Q(R(QR))) circuit (Fig 3b) was used to fit the data obtained for the alloys exposed to *S*. *mutans* species [19]. Q is a constant-phase element (CPE) that represented a shift from the ideal capacitor and was utilized instead of the capacitance to take into account the real capacitive behavior of the film. By using the R(QR) element, it was assumed that the oxide layer consisted of a compact layer with polarization resistance R_1_ and capacitance CPE_1_. Thus, R(Q(R(QR))) represented a biofilm formation model, in which R_1_ was the alloy charge transfer resistance, R_2_ was the biofilm resistance, CPE_1_ was the alloy capacitance, and CPE_2_ was the capacitance of the biofilm layer. In both circuits, R_s_ corresponded to the electrolyte resistance. Gunasekaran et al. [19] used the same circuits to fit various data. In this study, R(QR) was successfully utilized to fit the data obtained for the AS mixture without *S*. *mutans*, whereas R(Q(R(QR))) was found to be more suitable for describing the biofilm properties in the AS containing *S*. *mutans*.

**Fig 4:**
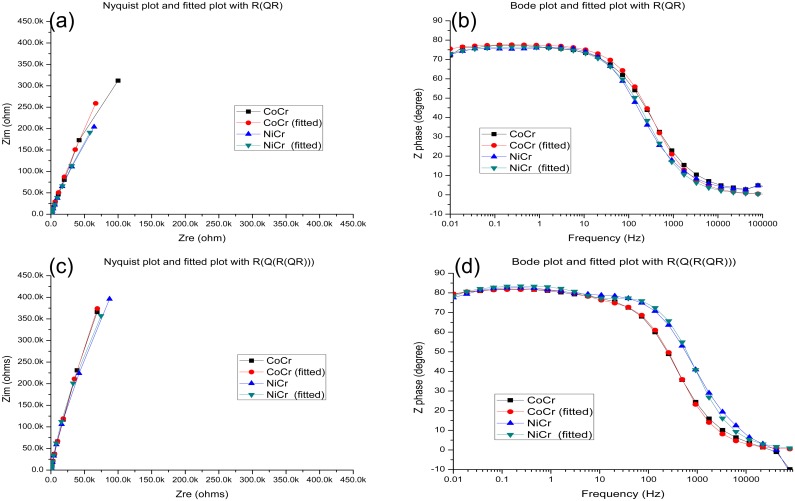
(a) A Nyquist plot fitted using the R(QR) circuit. (b) A Bode plot fitted using the R(QR) circuit. (c) A Nyquist plot fitted using the R(Q(R(QR)))) circuit. (d) A Bode plot fitted using the R(Q(R(QR)))) circuit.

At high frequencies, the phase angle shift was almost 0°, and the ohmic resistance dominated the impedance [17]. Hence, it could be concluded that the alloys exposed to the simulated oral environment were in the passive state. The Nyquist and Bode diagrams fitted using different equivalent circuits (Fig 4) indicate that all the studied samples exhibited capacitive behavior in the frequency range between 10^−2^ and 10^5^ Hz, demonstrating very good agreement between the fitted and the experimental data (the corresponding chi-square values were below 1×10^−3^).

Table 4 lists the parameters obtained from the fitting procedure. The EIS results for the alloys treated with the sterile medium were fitted using the R(QR) circuit, whereas the alloys exposed to the medium containing *S*. *mutans* were fitted using the R(Q(R(QR))) circuit. The obtained data indicate that the alloy surfaces contained passive films, which were more homogeneous and compact in the absence of *S*. *mutans* species. In the presence of *S*. *mutans*, the studied alloys exhibited two time constants, and their parameters were more consistent with those of the R(Q(R(QR))) circuit, suggesting the formation of porous films on the alloy surfaces and thus the creation of biofilms on the surfaces of dental alloys. The solution resistance R_s_ was relatively low because of the presence of various salts in the utilized medium. The measured biofilm resistances suggest that the impedance first increased because of the biofilm formation and then decreased after the biofilm desorption.

**Table 4:** Impedance parameters obtained for the two alloys immersed in the AS solutions with and without *S*. *mutans* species.

Alloy	Medium	R_s_ (Ω)	Q_1_ (CPE)		R_1_ (kΩ)	Q_2_ (CPE)		R_2_(Ω)
			Yo_1_(Ω^-1^ cm^-2^ s^n^)	n_1_		Yo_2_(Ω^-1^ cm^-2^ s^n^)	n_2_	
**CoCr**	Without *S*. *mutans*	37.9± 10.35	3.25±0.57 (×10−^5^)	0.891±0.025	5483± 1320	–	–	–
**CoCr**	With *S*. *mutans*	31.99±2.46	0.52±0.14 (×10−^5^)	0.973±0.025	7443± 2459	2.46±0.58 (×10−^5^)	0.923±0.011	4411± 2169
**NiCr**	Without *S*. *mutans*	66.23±40.50	5.37±0.74 (×10−^5^)	0.854±0.015	2537± 326	–	–	–
**NiCr**	With *S*. *mutans*	21.16±6.08	1.01±0.47 (×10−^5^)	0.970±0.030	2710± 570	2.88±0.90 (×10−^5^)	0.915±0.015	2440± 1234

### Bacterial surface adhesion

**Fig 5:**
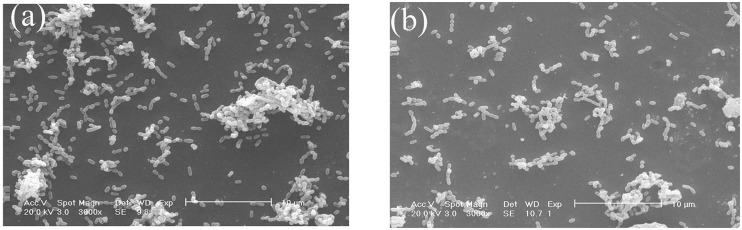
SEM images of the (a) CoCr and (b) NiCr alloy specimens exposed to the mixture of AS with *S*. *mutans* species for 24 h (magnification: 3, 000×).

The adherence of *S*. *mutans* species to the CoCr and NiCr dental alloy surfaces treated with Solution 2 for 24 h is illustrated by the SEM images depicted in Fig 5a and 5b, respectively (relatively large clumps of cells can be observed at a magnification of 3 000×). In these images, *S*. *mutans* bacteria both adhere to the specimen surfaces and overlap with each other, while the resulting biofilm appears to be discontinuous. The obtained SEM results are consistent with the EIS data reported in the previous section, indicating that the presence of microorganisms is able to drastically change the electrochemical conditions at the metal/solution interface due to biofilm formation.

### Potentiodynamic polarization measurements

**Fig 6:**
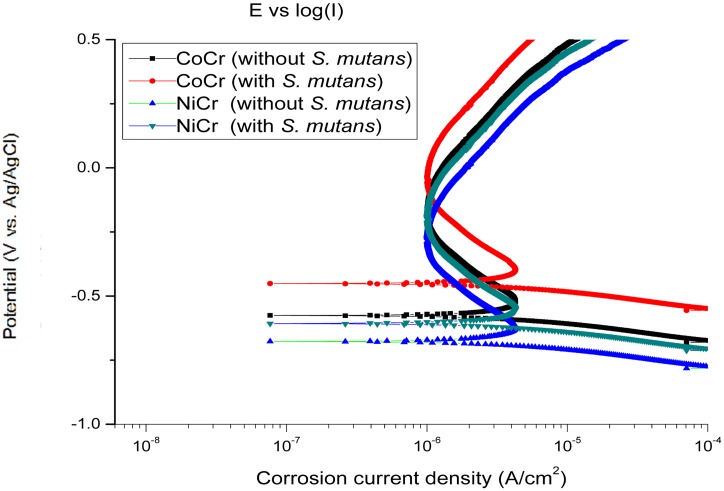
Typical potentiodynamic polarization curves obtained for CoCr and NiCr alloys treated with Solutions 1 and 2.

The potentiodynamic polarization curves recorded for CoCr and NiCr alloys immersed in Solutions 1 and 2 for 24 h are shown in Fig 6. The icorr values measured for the samples exposed to *S*. *mutans* were slightly lower than the magnitudes obtained after treatment with Solution 1, and their corresponding OCPs (vs. Ag/AgCl) were shifted to the positive direction (Fig 1).

Table 5 lists the OCP (vs. Ag/AgCl) and i_corr_ values for the dental alloys immersed in the two electrolytes. The i_corr_ of the NiCr alloy was higher than that of the CoCr alloy, which suggests that the former material is more prone to corrosion in the AS medium with or without *S*. *mutans*.

**Table 5:** OCPs (vs. Ag/AgCl) obtained from Fig 2 and icorr values estimated from the potentiodynamic polarization studies of CoCr and NiCr alloys treated with Solutions 1 and 2 (x±s).

Alloy	Solution	OCP (vs. Ag/AgCl) (mV) (error)	icorr (μA/cm^2^) (error)
**CoCr**	AS without *S*. *mutans*	–175.25 (±37.05)	0.195 (±0.032)
**CoCr**	AS with *S*. *mutans*	–237.50 (±14.39)	0.110 (±0.013)
**NiCr**	AS without *S*. *mutans*	–244.50 (±25.01)	1.683 (±0.160)
**NiCr**	AS with *S*. *mutans*	–274.25 (±18.32)	1.540 (±0.211)

## Discussion

This study was performed to investigate the dependence of the corrosion behavior of two dental alloys on the presence of *S*. *mutans* bacteria. An experimental medium for growing bacterial species was created to reproduce the human oral environment as closely as possible. All the utilized instruments used were carefully sterilized before each experiment, and particular attention was paid to preventing contamination due to external pollution.

When metals are immersed in test solutions, electrochemical reactions occur at the metal-solution interface. In this study, the OCPs of the tested samples exhibited negative shifts in the presence of *S*. *mutans*. Chang et al. [9] obtained similar results for pure Ti metal, Ti alloys, and Ni–Ti alloys exposed to *S*. *mutans*, which suggested that *S*. *mutans* or their byproducts produced during the corrosion process affected the OCPs of the tested samples at the metal-solution interface due to the formation of an oxide layer.

A potential (vs. Ag/AgCl) drop was observed for the CoCr and NiCr specimens after their exposure to *S*. *mutans* species, which could be related to their metabolism. The data obtained for both alloys exposed to *S*. *mutans* bacteria were consistent with the results of previous studies [20–25]. However, OCP measurements alone do not provide sufficient information for the accurate evaluation of the corrosion resistance of metals [26].

All samples examined in this study exhibited high impedance at low frequencies, suggesting the existence of a thin oxide passivation layer on the alloy surface, characterized by high corrosion resistance. When an alloy was immersed in the AS containing *S*. *mutans* for 24 h, biofilm formation occurred, which prevented charge transfer from the metal surface and thus decreased the alloy corrosion rate. Because all the studied samples demonstrated high impedance at low frequencies, it could be concluded that the alloys containing thin oxide passivation layers were characterized by high corrosion resistance in the specified medium [27,28]. The biofilm layer formed on the sample surface may act as a barrier that inhibits ionic conductivity or product diffusion [29].

The obtained SEM images showed that *S*. *mutans* species adhered to the sample surfaces and overlapped with each other, and that the biofilm formed by the bacteria was discontinuous. According to the EIS results, the charge transfer resistance of the two alloys exposed to *S*. *mutans* was higher than that of the alloys in the sterile medium. The icorr values measured for all samples decreased in the presence of *S*. *mutans* because of the absence of oxygen species from the metal-solution interface, indicating that the corresponding corrosion rate was also low. According to Chang et al. [9], the observed phenomenon can be explained by the ability of the oxide layer to resist attacks of *S*. *mutans* bacteria. Videla [30] suggested that corrosion inhibition was achieved due to the activity of sessile bacterial cells that formed a protective film and thus impeded the diffusion of corrosion products from the metal surface. To inhibit corrosion, it is necessary to localize the present nutrients species and thereby enhance the metabolic activity of the surface-assisted bacterial cells. When a sample was immersed in the AS mixture with *S*. *mutans* species, the microorganisms that adhered to the sample surface formed a biofilm, that prevented the charge transfer process from occurrence (in other words, it increased the alloy corrosion resistance) [9,31]. The resulting protective biofilm barrier prevented the diffusion of surface corrosion products, thus inhibiting the entire corrosion process [31,32].

The resulting biofilms composed of *S*. *mutans* monocultures were not only transformed from bacterial clusters into discrete layers, but also characterized by the increased thicknesses and heterogeneity. Moos et al. [33] showed that the microorganism growth on the metal surface leading to biofilm formation could affect the metal corrosion process by producing a gel phase which acted as a diffusion barrier and created concentration cells for metabolic and corrosion products. The authors of the described study also suggested that their results were applicable only to the well-known forms of corrosion and not to any new corrosion types. The produced biofilms (which contained differential aeration cells) caused local variations of the amounts of metabolic products, pH values, and dissolved oxygen levels.

By affecting the types and concentrations of the present ions (in addition to the pH and oxygen levels), the microorganisms induce significant variations in the physical and chemical characteristics of the environment as well as the electrochemical parameters utilized for measuring corrosion rates [34]. They affect metal corrosion resistance by changing the chemistry of the electrolytic solution on the metal surface. The generally accepted explanation of the microbial corrosion mechanism is that it begins with the formation of a biofilm on the metal surface because of the activity of aerobic microorganisms under favorable conditions [35]. Once the corrosion process is initiated, it is common to observe intense microbiological activity near corrosion sites. Direct counting of the organisms present in the bulk electrolyte is not sufficient for predicting their influence on the metal corrosion behavior; only the microorganisms that are in direct contact with metal surfaces affect metal corrosion properties. Videla et al. [34] noted that biofilms on alloy surfaces are usually formed within the first 24–72 h after the metal exposure to a bacteria-containing environment. The present microorganisms most likely do not participate directly in the corrosion process, but rather change the interfacial environment by creating concentration cells that facilitate corrosion resistance [36].

When the immersion time increases, the bacterial count increases, thus preventing corrosion by forming a protective layer on the alloy surface. Gunasekaran et al. [19] reported a significant reduction in the corrosion rate observed in the presence of *Pseudomonas flava* bacteria. The protective film formation began within the first 24 h of treatment and continued until 96 h have passed after which the produced film began to deteriorate. Therefore, the long-term effects of *S*. *mutans* on the corrosion behavior of CoCr and NiCr alloys should be investigated in detail in future studies.

## Conclusions

In this work, the corrosion properties of NiCr and CoCr alloys immersed in the AS solution containing *S*. *mutans* for 24 h have been examined. The obtained EIS results confirmed that the biofilm formation played an important role in the alloy corrosion process, while the presence of *S*. *mutans* in the solution reduced the corrosion rate of the studied alloys. In particular, the protective biofilms formed on the surfaces of dental alloys were capable of inhibiting the corrosion process by acting as physical barriers to oxygen attacks. They did not participate directly in the corrosion reaction, but rather changed the interfacial environment by creating concentration cells facilitating corrosion resistance. The obtained results indicate that microbiologically influenced corrosion inhibition is a more common phenomenon than has been previously assumed.

## Supporting information

S1 File Raw data of Figs 1, 2, 4 and 6. (XLSX)

S2 File Raw data of Fig 1. (MDB)

S3 File Raw data of Figs 2 and 4. (MDB)

S4 File Raw data of Fig 6. (MDB)
